# Electrochemically stimulating developments in bioelectronic medicine

**DOI:** 10.1186/s42234-018-0001-z

**Published:** 2018-03-15

**Authors:** Paola Sanjuan-Alberte, Morgan R. Alexander, Richard J. M. Hague, Frankie J. Rawson

**Affiliations:** 1 Regenerative Medicine and Cellular Therapies, School of Pharmacy, University of Nottingham, Nottingham, NG7 2QL UK; 2 Centre for Additive Manufacturing, School of Engineering, University of Nottingham, Nottingham, NG7 2QL UK; 3 Advanced Materials and Healthcare Technologies, School of Pharmacy, University of Nottingham, Nottingham, NG7 2QL UK

**Keywords:** Bioelectronic interfaces, Bioelectrochemistry, Nanobioelectronics, Cellular signalling

## Abstract

Cellular homeostasis is in part controlled by biological generated electrical activity. By interfacing biology with electronic devices this electrical activity can be modulated to actuate cellular behaviour. There are current limitations in merging electronics with biology sufficiently well to target and sense specific electrically active components of cells. By addressing this limitation, researchers give rise to new capabilities for facilitating the two-way transduction signalling mechanisms between the electronic and cellular components. This is required to allow significant advancement of bioelectronic technology which offers new ways of treating and diagnosing diseases. Most of the progress that has been achieved to date in developing bioelectronic therapeutics stimulate neural communication, which ultimately orchestrates organ function back to a healthy state. Some devices used in therapeutics include cochlear and retinal implants and vagus nerve stimulators. However, all cells can be impacted by electrical inputs which gives rise to the opportunity to broaden the use of bioelectronic medicine for treating disease. Electronic actuation of non-excitable cells has been shown to lead to ‘programmed’ cell behaviour via application of electronic input which alter key biological processes. A neglected form of cellular electrical communication which has not yet been considered when developing bioelectronic therapeutics is faradaic currents. These are generated during redox reactions. A precedent of electrochemical technology being used to modulate these reactions, thereby controlling cell behaviour, has already been set. In this mini review we highlight the current state of the art of electronic routes to modulating cell behaviour and identify new ways in which electrochemistry could be used to contribute to the new field of bioelectronic medicine.

## Background

Bioelectronic medicine is typically conceptualised as electronic technology that merges with neurons enabling control of cellular electrical communication and the underlying organ function. Electrical communication that is mediated by neurons is the body’s universal fast electrical communication system that orchestrates organ function at a macro level. The underlying principles of the body’s electrical communication system, from a traditional point of view, originates from the controlled bulk movement of ions across the plasma membrane of cells. This enables the establishment and modulation of membrane potentials and in doing so, produces an electrochemical potential gradient that can drive bulk charge movement. The movement of charges across the membrane lead to action potentials, which represent a major electrical communication route in muscle cells, neurons and endocrine cells (Loewenstein 1981). Other function of the membrane potential is the transport of molecules across it, induced by the translocation of charges or electrogenic transport. This includes the transport of molecules such as glucose, ATP and small peptides involved in a plethora of physiological roles (Sundelacruz et al. 2009; Rothbard et al. 2004; van Horssen et al. 2005; Franco et al. 2006).

**Fig. 1:**
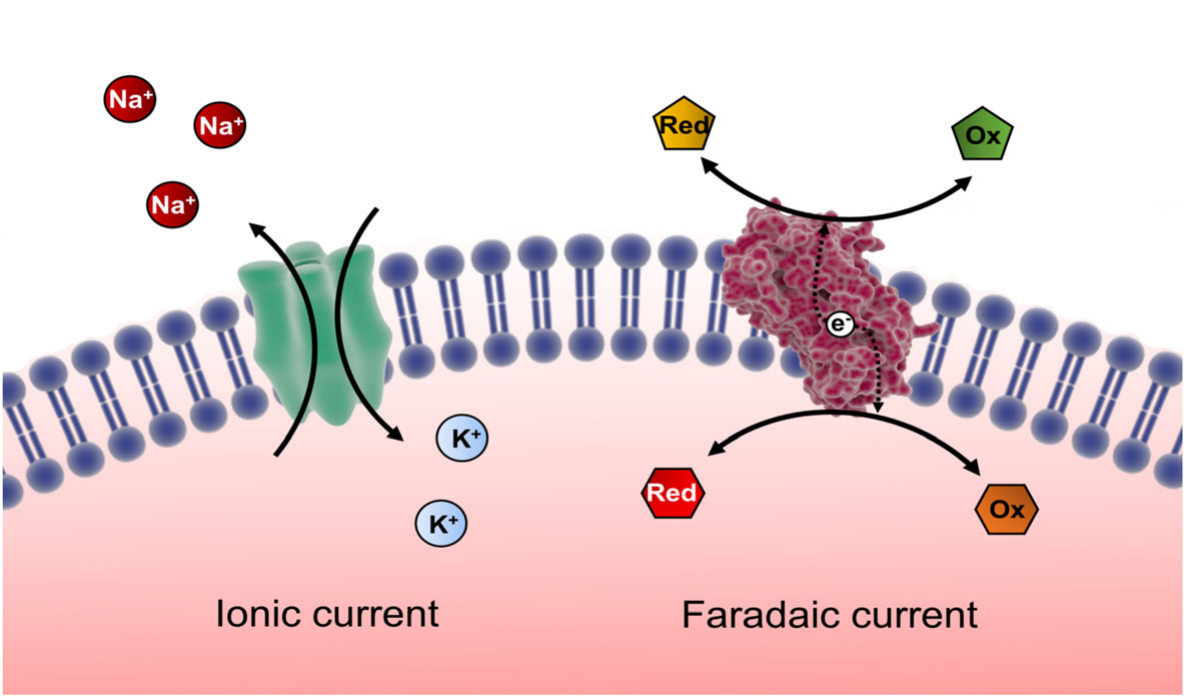
Bulk ion vs faradaic conductance across the cell plasma membrane. Ionic currents are produced by the movement of charges across the membrane through the ionic channels, whereas faradaic currents are produced by the movement of electrons between electrochemical mediators

Importantly, all cells also use faradaic currents besides other communication routes (Boyd et al. 2014) to communicate with one another and are vital for maintaining homeostasis. Biochemical processes that result in the production of faradaic current, which is defined as the movement of electrons, are generated in redox reactions. In order for redox reactions to happen, an exchange of electrons between two biochemical entities, an electron donor and an electron acceptor, needs to be produced (Fig. 1). Faradaic currents are reliant on naturally occurring biological electrochemical mediators, which are defined as molecules that readily accept or donate an electron(s), and some of the most well known and some of the most abundant are NADH, NADPH, GSH, Ascorbic acid and ubiquinone. Additionally, biomacromolecules such as enzymes (for example oxido-reductase) can also act as electron shuttles. Examples of faradaic signalling include the generation of oxidant sources within the mitochondrial respiratory chain in response to bacterial infection or inflammation (Holmstrom and Finkel 2014). Moreover, all cells use membrane electron transport systems to shuttle electrons across membranes for a wide range of purposes. The electron transport via membrane bound systems have been implicated in cell signalling, nutrient metabolism, cell redox maintenance and can play an important role in disease such as cancer. Opportunities arise when we substitute a biological electrochemical mediator, involved in generating electrical faradaic current, with electrodes. This yields the ability to control redox events when interfacing cells with electronics by modulating the electron flow in a specific biochemical event via applied electrical potential stimulus, as a result leading to modulation of the underlying biochemistry.

Despite their extensive use in cellular sensing, faradaic processes have been largely neglected when considering the state of play and design of bioelectronic methods now used for disease intervention. This largely unexplored view of cellular electrical communication, from the perspective of developing new bioelectronic devices, offers new opportunities in modulating cell state and therefore underlying cells, tissues and organ function. The aim of this mini-review is to place into context how this faradaic form of cellular electrical communication could be used to develop bioelectronic medicines. In addition, this review aims to detail the early examples of such technology that can interface with cells to both sense and modulate cell behaviour.

## Development of bioelectronic therapies

The concept of bioelectronic medicine consists of merging biological systems with electronic devices, allowing for the modulation of underlying cellular, tissue and ultimately organ function by regulating bio-electrical communication via electrical input from the electronic device. In order to achieve efficient communication between biological and electronic systems, transduction of signals at the cellular-electronic interface must be achieved (Carrad et al. 2016). This requirement presents a key challenge in developing bioelectronics and is currently being explored to advance the progression of bioelectronic devices with therapeutic interest (Zhang and Lieber 2016). An obstacle to this is that the building blocks of cellular structures differ from those that can be found when constructing electronics, meaning that the seamless integration of electronics with biology is not yet possible. In addition, the plasticity of young nervous systems and tissues present a key obstacle as implantable electronics cannot adapt and therefore need regularly servicing. Advancement in manufacturing technology will aid in yielding a solution to this problem of overcoming biological  plasticity. For example, additive manufacturing techniques are particularly appealing for the production of novel three-dimensional bioelectronic tools, allowing a synergistic integration of electronic components with the biological building blocks (Kong et al. 2016). This is due to their capability of combining conductive materials and living cells in unique architectures, resulting in functional devices (Ladd et al. 2013; Mannoor et al. 2013). Therefore, there are technical challenges in the field which require solutions from a combination of experts which include physicists, chemists, engineers and biologists.

However, significant progress has been made with several electronic devices successfully introduced as therapeutics aimed at palliating disabilities, and consequently become good examples of an effective transduction of signals. Examples include known cochlear or retinal implants where sound and light, respectively, are converted into electrical signals that can be transmitted to the nervous system and interpreted by the patient’s brain (Heiduschka and Thanos 1998; Humayun et al. 2016).

**Fig. 2:**
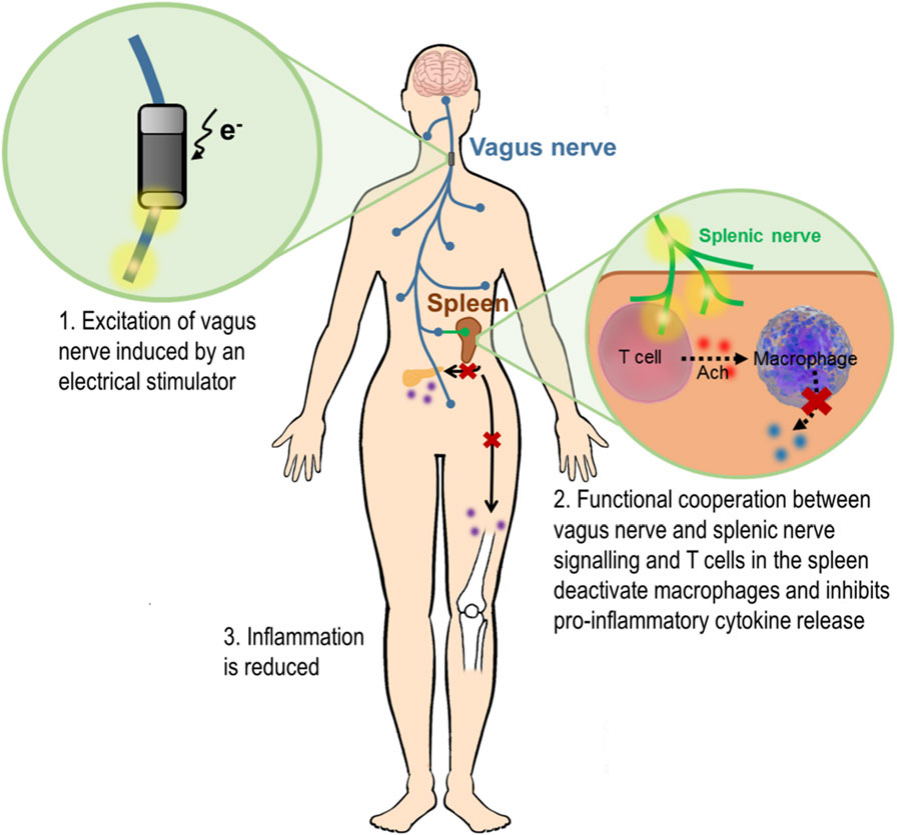
Schematic representation of a bioelectronic approach at targeting the vagus nerve to control inflammation. Vagus nerve signalling interacts with the splenic nerve that reaches splenic T cells that produce acetylcholine, which reduces inflammation

Stimulation of nerve fibres based on direct vagus nerve stimulation is also gaining interest as a treatment of rheumatoid arthritis and metabolic syndrome (Pavlov and Tracey 2012; Koopman et al. 2016; Famm et al. 2013) (Fig. 2). This therapeutic strategy demonstrates the influence of the nervous system over the different biological functions and disease states. Further investigation in this subject can possibly help in decoding the neural circuits and informing how nerve stimulation correlates to the homeostatic state of an organism, leading to new means of treating and diagnosing disease. However, to achieve this a higher resolution in terms of electrical targeting of specific cells and tissues is needed. Similar devices based on the depolarisation of the sinus node to induce action potentials can be found for excitation of cardiac cells in the therapy of heart failure and atrial fibrillation (Dobrzynski et al. 2007).

Currently electrical stimulation with these devices is unable to target cells individually, and lack high resolution in terms of targeting, inducing the excitation of the whole tissue. Due to the highly compact state of the nervous and cardiac systems, indiscriminate stimulation can lead to undesirable effects or mire the clinical outcomes. Application of inputs on specific cells would be beneficial to achieve a fine degree of regulation. Advances in nanotechnology have contributed to the development of structures such as nano-field effect transistors (nano-FETs) or nanowires (NWs) capable of stimulate and record signals from individual cells, increasing the prospects of targeting intracellular components (Xie et al. 2015; Gao et al. 2012). It is therefore envisaged that future advancements in this technology will aid in the development of bioelectronic therapeutics aimed at increasing the selectivity and specificity of cellular control.

## Reaching non-excitable cells

**Fig. 3:**
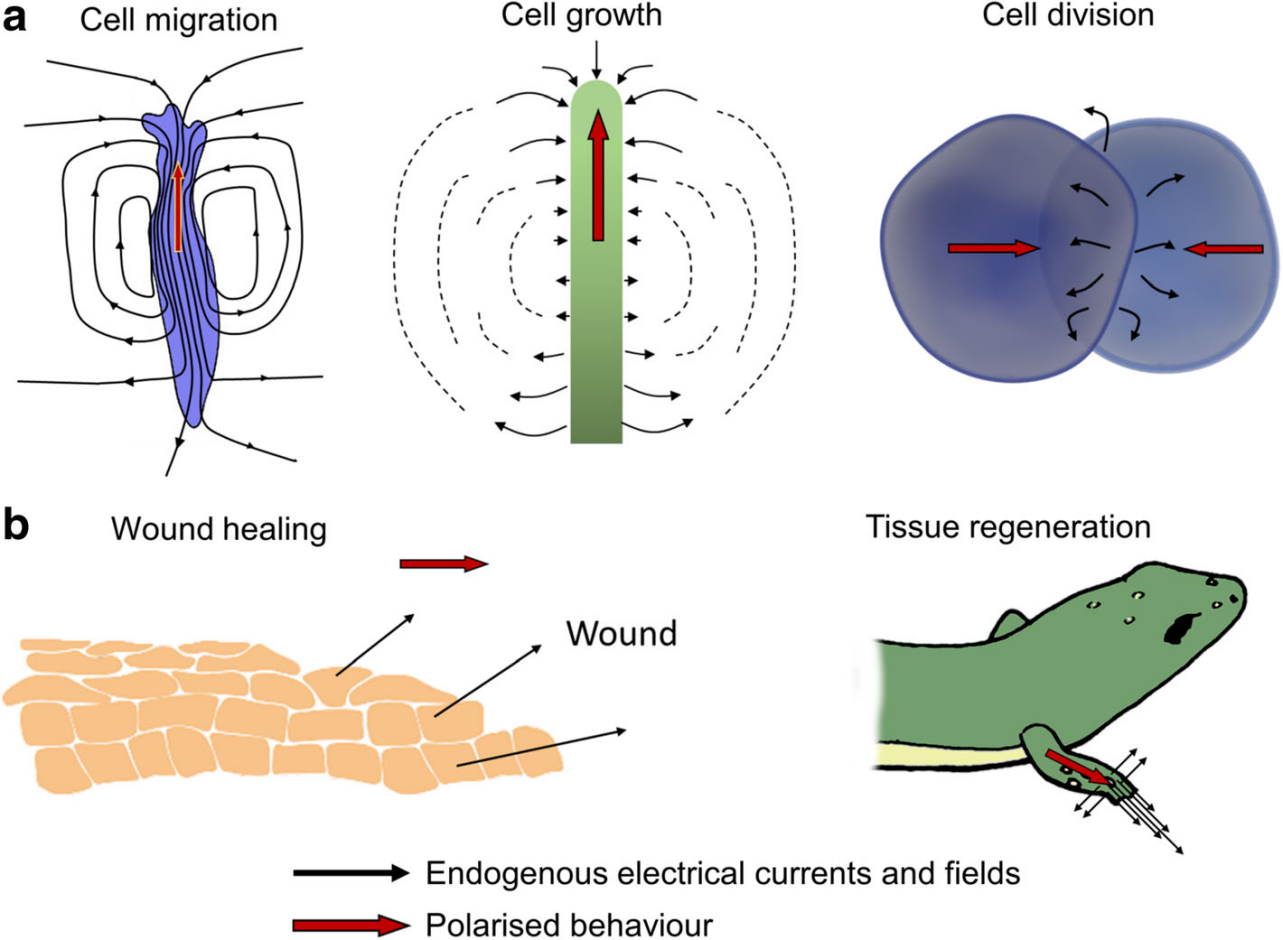
Different biological functions can be triggered by the application of electric fields and these include actuating cell movement, modulating the cell cycle which benefits wound healing and tissue regeneration. Currents can polarise a) single cells and b) tissues. Black arrows indicate the direction of the electrical currents whereas red arrows indicate the direction of the resulting polarised behaviour (Chang and Minc 2014)

Cells that are unable to propagate action potentials, known as non-excitable cells, also possess electrical properties and endogenous electric fields to direct growth or healing (Levin 2014). Interfacing these cells with electronic devices gives rise to new opportunities that should allow for the control of cellular function. This natural bioelectric behaviour can be organised and stimulated by applying electric currents that control the polarisation of the membrane potential. This induced transmembrane potential can regulate the passage of molecules and ions across it by, for instance, controlling Ca^2+^ and epidermal growth factor receptor (EGFR) channels (Li et al. 2017). Defective ion and molecular transport have implications on diverse diseases such as cancer or pulmonary oedema (Prevarskaya et al. 2010; Hollenhorst et al. 2011). Therefore, achieving good degree of control of transport across the cellular membrane could have great repercussions in the treatment of these diseases. Application of exogenous electric fields has been demonstrated to be effective in both single cells and tissues, triggering a wide range of biological actions (Chang and Minc 2014) (Fig. 3).

Due to the high resistance of the cellular membrane to current flow, ionic currents induced by artificial external electric fields are forced to surround the cells, imposing a potential gradient across the membrane surface. This potential gradient induces changes in function and/or orientation of membrane proteins and opening of ionic channels, leading to stimulation of intracellular signalling pathways (Yao and Li 2016). The signalling cascade alters expression of genes which code for proteins involved in several biological functions including cell division, migration, proliferation and embryogenesis.

The gap junctions between cells, which are channels connecting cellular cytoplasms, also have an important role when applying electric fields in order to generate a response at a tissue level. This slow communication route can amplify the intracellular signalling cascade produced in response to the changes in the potential (Levin 2014). Signals propagate through the tissue, triggering a coordinated cellular response to, for example, wound healing or tissue regeneration. This approach has been introduced in the therapy of bone fracture healing and osteoarthritis, stimulating the chondrocyte and osteoblast regeneration. As a result, osteogenesis and increases in bone mineral density was observed (Maziarz et al. 2016).

Cancer therapy can also be benefited from the use of electric fields. There have been many studies reporting the differences in resting membrane potentials between tumour and non-tumour cells. Generally, it can be established that cells with a high proliferative activity such as embryonic, stem and metastatic cells possess a depolarised membrane (Binggeli and Weinstein 1986). Metastatic phenotypes can be induced in healthy cells by depolarisation of their membrane and conversely, the activity in a metastatic cell induced by oncogenes can be supressed by preventing its depolarisation (Levin 2014).

Additional research in this area will allow a deeper understanding of the precise mechanisms involved, which may make it possible to program cellular activity via the use of electric fields. Importantly this broadens the prospective applications of bioelectronic medicine beyond neural control.

## Improving electronic targeting: an electrochemical approach

In order to create bioelectronic tools with such capabilities in cell programming, further specificity on the cellular outputs is desirable. Biomolecular entities, including redox biomolecules, are known to be highly specific as they represent a transfer system of biological information. Therefore, controlling faradaic currents involved in cellular redox reactions offers opportunities for the electrochemical mediated induced control of cells, tissues and organ function.

**Fig. 4:**
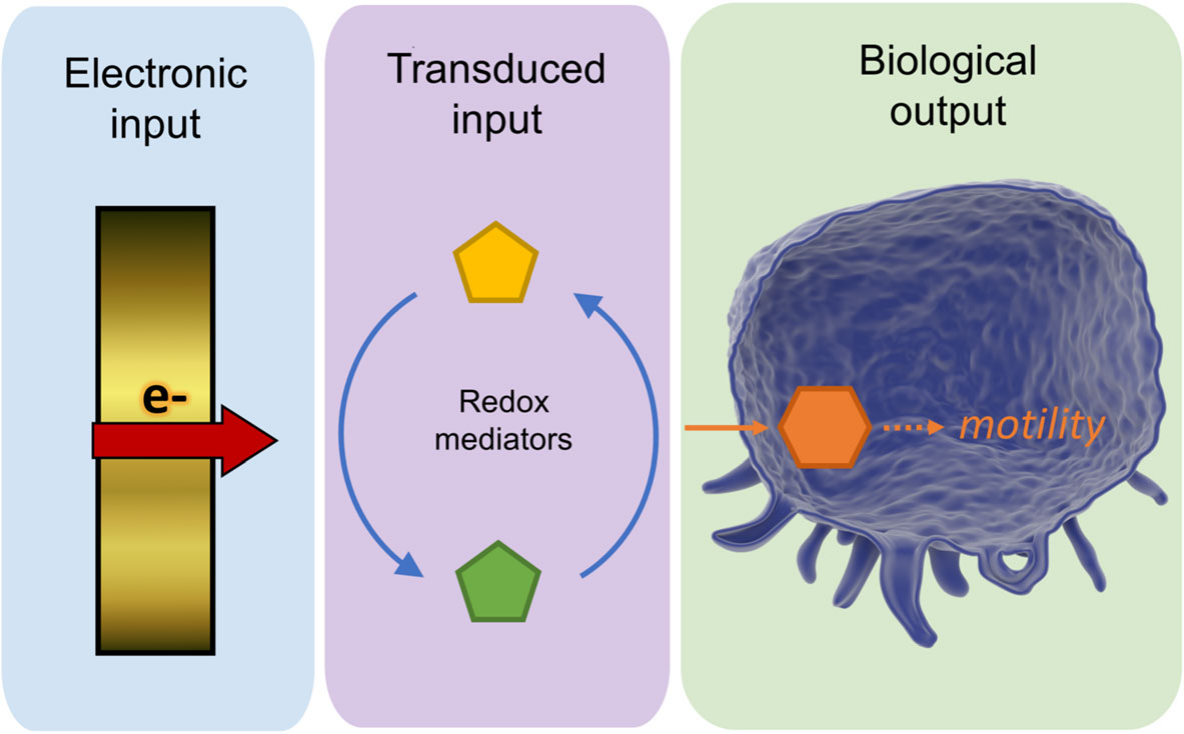
Transduction of signals on a bioelectrochemical system. An electronic input in the form of potential modulates the redox state of naturally occurring electrochemical mediators, from an inactive state to an active state or vice versa*,* and are communicated to a cellular system triggering biological response (Tschirhart et al. 2017)

The faradaic processes can be hijacked by modulation of polarity or by using electrochemically active molecules. Electronic inputs are transduced into redox active mediators that ultimately activate a biological mechanism (Fig. 4). Biological responses often correlates with the magnitude, frequency, and/or type of electronic input applied (Gordonov et al. 2014), indicating that a fine degree of control can be achieved. For this reason, the authors believe that bioelectrochemical devices with ability to control cell function and disease state can be included in the field of bioelectronic medicine (Rawson 2015).

Formation of effective interfaces between electrodes and cells is possible by engineering the electrode surface at the nanoscale. This gives rise to potential for sensing and controlling faradic processes and has been reviewed recently (Du et al. 2013; Ajo-Franklin and Noy 2015). In general, approaches to electrically ‘wire’ cell redox components rely on electrode modification with conducting polymers (Saboe et al. 2016), nanowires based on carbon nanofibers (Rawson et al. 2016), carbon nanotubes (Rawson et al. 2012; Gooding et al. 2003) and electrocatalysts (Rawson et al. 2015). This wiring can also be achieved via modification of electrode surfaces with chemical entities that bond to the saccharide groups of the eukaryotic cells that facilitate electron transfer (Stephenson-Brown et al. 2015). In addition, structures integrated with biological components such as enzymes, lipid bilayers or antibodies are used to transduce ions into electrical currents and vice-versa for recording and stimulation of biological reactions in both intracellular and extracellular environments (Strakosas et al. 2017). For instance, electrodes can be conjugated with neurotransmitters to induce neural excitability (Simon et al. 2009) or regulate pH using protonic devices to control enzymatic function, and acid sensitive ion channels (Strakosas et al. 2017).

## Future opportunities in directing cell behaviour electrochemically

Reactive oxygen species (ROS) may be used to direct cell function. ROS are involved in signalling pathways that take part in several biological events associated to bacterial infection and cancer. The production of ROS can be controlled by electrochemical generation (Rawson et al. 2015). Gene transcription can be induced in response to oxidative stress, inducing cell motility or cell-to-cell communication (Tschirhart et al. 2017). Therefore, electrochemical control of ROS generation may prove fruitful for directing cell behaviour.

Further development of bioelectrochemical devices may have great implications in future cancer therapy by individually controlling plasma membrane electron transport systems (tPMETs). The system of tPMET ferri-reductase is upregulated and it is thought to enable faster rates of metabolism in cancer cells (Rawson 2015). Therefore, electrochemical tools with capacity to control such systems may regulate metabolism and cellular development.

## Conclusions

Bioelectronic medicine is a growing field where major advancement in treatment and diagnosing of diseases are being achieved. Therapies based on neural stimulation and application of electric fields are currently used to improve patient’s quality of life, but additional control of the effects is still required.

The main challenges include creation of effective biological-electrical interfaces and transduction of signals. In order to modulate the electron transfer events, an intimate contact of the electronic component with the active sites is required. Therefore, technological advancements in the interfacing of electronics with such active sites are necessary to fully integrate biological systems and electronic devices.

Further specificity can also be achieved by controlling redox biomolecules and the biological output with great precision, adding new proportions to the bioelectronic medicine field. However, bioelectrochemical therapies still require a multidisciplinary approach to produce less invasive techniques, e.g. using wireless systems. In order to achieve this, development of nanotechnology, materials and new methodologies will greatly contribute to this field offering new therapeutic tools.

A more thorough understanding and controlled targeted stimulation of vagus nerve, in addition to ROS production could be used to control of inflammatory mediators that take part in diseases such as artherosclerosis, pulmonary fibrosis, Parkinson’s disease and Alzheimer’s disease. Cancer therapy can also be impacted by development of bioelectrochemical systems to direct tPMET activity, regulating cellular behaviour.

It can thus be concluded that this field has many open paths and offers many exciting approaches and research opportunities that will contribute to create great impact over the future medicine and pharmacology.
